# Pharmacological regulation of chondrocyte ferroptosis by plant-derived metabolites in osteoarthritis: from molecular mechanisms to targeted nanodelivery

**DOI:** 10.3389/fphar.2026.1949976

**Published:** 2026-10-09

**Authors:** Tianci Guo, Qi Liu, Jida Wang, Keming Ren, Longyao Zhang, Jinzhong Sheng, Aifeng Liu

**Affiliations:** 1 First Teaching Hospital of Tianjin University of Traditional Chinese Medicine, Tianjin, China; 2 National Clinical Research Center for Chinese Medicine, Tianjin, China

**Keywords:** osteoarthritis, chondrocyte, ferroptosis, plant-derived metabolites, pharmacology, nanodelivery

## Abstract

Osteoarthritis (OA) lacks approved disease-modifying pharmacotherapy. Chondrocyte ferroptosis—iron-dependent phospholipid peroxidation caused by disrupted iron handling, lipid metabolism, and antioxidant defense—has been implicated in cartilage degeneration and is pharmacologically suppressible in experimental models. Natural products can modulate multiple ferroptosis-related nodes, including the Nrf2/SLC7A11/GPX4 axis, ACSL4-dependent lipid peroxidation, iron homeostasis, and mitochondrial redox balance. Their translation, however, is limited by poor solubility, instability, rapid clearance, uncertain dose–exposure relationships, and restricted cartilage penetration. This review integrates the pharmacological basis of chondrocyte ferroptosis, evidence for anti-ferroptotic natural products, and nanodelivery strategies that improve joint retention, cartilage targeting, chondrocyte uptake, and controlled release. We distinguish direct ferroptosis evidence from adjacent antioxidant or chondroprotective findings and evaluate polymeric, lipid-based, hydrogel, biomimetic, inorganic, and hybrid platforms as pharmacological optimization tools. Current integrated systems show encouraging structure-preserving effects in experimental OA, but evidence remains predominantly preclinical. Translation will require standardized compounds, ferroptosis-specific target-engagement assays, pharmacokinetic and dose–response studies, carrier-specific safety testing, clinically relevant models, and biomarker-guided patient selection. Rationally matching natural-product pharmacology with joint-specific delivery may enable mechanism-based OA therapies.

## 1 Introduction

Osteoarthritis (OA) is the most prevalent degenerative joint disease worldwide and a leading cause of chronic pain and disability, affecting an estimated 595 million people in 2020, with prevalence projected to increase as populations age and obesity becomes more common (GBD, 2021 Osteoarthritis Collaborators et al., 2023). Rather than representing passive wear and tear, OA is now understood as an active disorder of the whole joint involving cartilage, synovium, subchondral bone, menisci, and periarticular tissues. Nevertheless, current pharmacotherapy remains predominantly symptomatic: analgesics and non-steroidal anti-inflammatory drugs can reduce pain but do not reliably halt structural progression, and no disease-modifying OA drug has yet received regulatory approval (Hunter and Bierma-Zeinstra, 2019). Identifying pharmacologically actionable mechanisms that connect chondrocyte injury to matrix destruction is therefore a major priority.

Ferroptosis—an iron-dependent form of regulated cell death driven by the accumulation of phospholipid peroxides—has emerged as a plausible contributor to OA progression (Dixon et al., 2012). It develops when iron-catalyzed radical propagation and acyl-CoA synthetase long-chain family member 4 (ACSL4)-dependent polyunsaturated phospholipid remodeling exceed the detoxification capacity of the antioxidant protection provided by solute carrier family 7 member 11 (SLC7A11)-mediated cystine uptake, glutathione (GSH), and glutathione peroxidase 4 (GPX4) (Stockwell et al., 2017). Chondrocytes are particularly susceptible because they are long-lived cells embedded in an avascular matrix and exposed to chronic mechanical and inflammatory stress. Human OA cartilage and experimental models show reduced GPX4 activity, iron accumulation, lipid peroxidation, and characteristic mitochondrial changes; pharmacological or genetic suppression of ferroptosis can attenuate chondrocyte loss and cartilage degeneration (Yao et al., 2021; Cao et al., 2023). These observations support ferroptosis as a mechanistically tractable target, while not yet establishing it as a clinically validated driver in all OA phenotypes.

Natural bioactive products provide a broad chemical space for ferroptosis-directed pharmacology. The network nature of ferroptosis favors compounds capable of acting at several nodes, and polyphenols, flavonoids, terpenoids, saponins, glycosides, and alkaloids have been reported to regulate iron handling, lipid peroxidation, mitochondrial homeostasis, and endogenous antioxidant defenses. Representative compounds such as theaflavin-3,3′-digallate, asperosaponin VI, and baicalin suppress ferroptosis-related changes mainly through the nuclear factor erythroid 2-related factor 2 (Nrf2)/GPX4 pathway or inhibition of ACSL4-associated lipid peroxidation (Xu et al., 2022; Zhang et al., 2025; Liu et al., 2024). However, mechanistic promise does not automatically translate into therapeutic exposure. Poor aqueous solubility, chemical instability, extensive metabolism, rapid systemic or intra-articular clearance, and weak cartilage penetration often prevent free natural products from reaching sustained concentrations at the chondrocyte target.

Nanodelivery may bridge this pharmacological gap by improving apparent solubility and stability, prolonging joint residence, enhancing cartilage penetration and chondrocyte uptake, and enabling microenvironment-responsive release. Cartilage-binding ligands such as the type II collagen-binding peptide WYRGRL and redox- or pH-responsive materials can provide spatial and temporal control over drug exposure (Rothenfluh et al., 2008). Several experimental platforms have produced greater chondroprotection than the corresponding free compounds through combined effects on reactive oxygen species, GPX4 activity, inflammatory signaling, and extracellular matrix metabolism (Wang et al., 2025; Wang et al., 2026; Huang et al., 2025). Yet ferroptosis biology, natural product pharmacology, and carrier engineering have often been studied separately, making it difficult to determine which target, compound, and delivery design should be matched for a given OA context.

This review therefore provides a pharmacology-centered, design-oriented synthesis of the field. We first define the molecular mechanisms and druggable nodes through which ferroptosis may promote chondrocyte death and matrix catabolism, and then critically evaluate natural products according to their molecular targets, experimental models, evidence strength, and translational limitations. We subsequently examine the joint-specific transport barriers and nanodelivery principles required to convert mechanistically active compounds into therapeutically relevant intra-articular systems. By linking what should be modulated, which natural products can modulate it, and how those compounds should be delivered, we aim to identify current controversies, evidence gaps, and priorities for developing natural product-based disease-modifying OA therapies.

## 2 Pharmacological basis of chondrocyte ferroptosis in osteoarthritis

Ferroptosis is a non-apoptotic, iron-dependent form of regulated cell death executed by the unrestrained peroxidation of membrane phospholipids, and it is governed not by a single effector but by the dynamic equilibrium among three metabolic axes: cellular iron handling, polyunsaturated fatty acid (PUFA) metabolism, and the glutathione-dependent antioxidant defense (Jiang et al., 2021). Several features of articular chondrocytes render them intrinsically vulnerable to this death modality. They are long-lived, essentially post-mitotic cells embedded in an avascular matrix, where chronic mechanical loading and a comparatively limited antioxidant reserve predispose them to oxidative injury; moreover, the osteoarthritic joint is an iron- and cytokine-rich milieu that further tilts redox homeostasis toward lipid peroxidation (Jing et al., 2021a; Jing et al., 2021b). Consistent with this, human OA cartilage and animal models display the biochemical and ultrastructural hallmarks of ferroptosis—diminished GPX4, accumulation of ferrous iron and malondialdehyde, and shrunken, high-density mitochondria—predominantly within the mechanically loaded regions of the tissue, and the severity of these alterations tracks with histological disease grade (Wang et al., 2022; Xu et al., 2023a). Delineating how these axes are deranged in chondrocytes is therefore essential both to understand OA pathogenesis and to identify the nodes at which natural products and nanocarriers can be deployed (Gao et al., 2024). The following subsections dissect each axis in turn and then examine the self-amplifying circuit linking chondrocyte ferroptosis to extracellular matrix (ECM) destruction.

### 2.1 Disrupted iron homeostasis and expansion of the labile iron pool

Iron accumulation is the upstream prerequisite for chondrocyte ferroptosis, and the osteoarthritic microenvironment perturbs nearly every step of cellular iron handling. Pro-inflammatory cytokines abundant in the OA joint, notably interleukin-1β (IL-1β) and tumor necrosis factor-α (TNF-α), coordinately upregulate the iron-import proteins transferrin receptor 1 (TfR1) and divalent metal transporter 1 (DMT1) while suppressing the principal iron exporter ferroportin (FPN), driving a net influx of iron into chondrocytes. In parallel, ferritinophagy—the selective autophagic degradation of ferritin executed by the cargo receptor nuclear receptor coactivator 4 (NCOA4) (Mancias et al., 2014)—liberates stored iron and is markedly enhanced in OA cartilage; the JNK–JUN axis transcriptionally activates NCOA4, and its inhibition restrains chondrocyte ferroptosis and attenuates post-traumatic OA *in vivo* (Sun et al., 2023). The convergence of increased uptake and ferritin breakdown expands the redox-active labile iron pool, in which ferrous iron (Fe^2+^) fuels the Fenton reaction to generate hydroxyl radicals that ignite lipid-radical propagation; the attendant mitochondrial dysfunction can be mitigated by calcium chelation, underscoring the tight coupling of iron overload to organelle injury (Jing et al., 2021c). The pathogenic weight of this iron influx is reinforced by functional studies: experimental iron overload aggravates cartilage destruction and elevates the matrix-degrading enzymes MMP-13 and ADAMTS-5, whereas blockade of TfR1—for example with the selective inhibitor ferstatin II—restores iron homeostasis, suppresses oxidative stress and mtDNA–cGAS–STING-driven inflammation, and ameliorates cartilage degeneration, identifying the iron-import machinery as a tractable upstream target (Wang et al., 2024).

### 2.2 Lipid peroxidation as the executioner of chondrocyte ferroptosis

Whereas iron supplies the catalyst, the proximate executioners of ferroptosis are oxidized membrane phospholipids. Acyl-CoA synthetase long-chain family member 4 (ACSL4), acting together with lysophosphatidylcholine acyltransferase 3 (LPCAT3), esterifies PUFAs—chiefly arachidonic and adrenic acid—into membrane phospholipids, generating the oxidation-prone substrate pool that dictates ferroptosis sensitivity (Doll et al., 2017). These PUFA-containing phospholipids are then peroxidized both enzymatically, principally by lipoxygenases, and non-enzymatically through iron-catalyzed radical chain reactions, yielding phospholipid hydroperoxides whose accumulation disrupts membrane integrity and precipitates cell rupture (Yang et al., 2016). In chondrocytes, this cascade is amplified by disease-specific regulators: hypoxia-inducible factor 2α (HIF-2α, encoded by EPAS1), a master catabolic transcription factor that is elevated in OA cartilage and directly induces matrix-degrading enzymes (Yang et al., 2010), also lowers intracellular glutathione and heightens susceptibility to lipid peroxidation, such that supplementation with D-mannose suppresses ferroptosis and cartilage degeneration in a HIF-2α-dependent manner (Zhou et al., 2021). The centrality of ACSL4-shaped membrane composition makes the lipid-peroxidation axis a particularly attractive target for radical-trapping and Nrf2-activating natural products, as discussed in Section 3.

### 2.3 Impaired cystine uptake and GPX4-dependent antioxidant defense

The lethal accumulation of phospholipid hydroperoxides is normally limited by glutathione peroxidase 4 (GPX4), the principal enzyme that directly reduces these products to non-toxic alcohols using glutathione (GSH) as a cofactor; GSH synthesis in turn depends on cystine imported through the cystine/glutamate antiporter system xc− (light-chain subunit SLC7A11) (Yang et al., 2014). Genetic inactivation of Gpx4 is sufficient to trigger lethal ferroptotic tissue injury *in vivo*, establishing this enzyme as a central gatekeeper of the pathway (Friedmann Angeli et al., 2014). Failure along the system xc−–GSH–GPX4 axis promotes ferroptosis, and OA cartilage exhibits coordinated downregulation of SLC7A11 and GPX4 with GSH depletion (Miao et al., 2022). GPX4 also links ferroptosis to matrix catabolism: its loss sensitizes chondrocytes to oxidative death and activates MAPK/NF-κB signaling, whereas FOXO3 activation restores GPX4 and SLC7A11 and limits experimental OA (Miao et al., 2022; Zhao et al., 2023). The FSP1–coenzyme Q10 axis operates in parallel with GPX4 to trap lipid peroxyl radicals (Bersuker et al., 2019), and FSP1 reinforcement or coenzyme Q10 supplementation attenuated OA changes in GPX4-deficient mice. Nrf2 coordinates several components of this antioxidant network, including SLC7A11, GPX4, and heme oxygenase-1, explaining why Nrf2/GPX4 signaling is a frequent pharmacological target in anti-ferroptotic OA studies.

### 2.4 From chondrocyte death to matrix catabolism and synovial inflammation

Ferroptotic chondrocyte injury may contribute to matrix degradation and synovial inflammation, but direct evidence that chondrocyte ferroptosis is a specific driver of damage-associated molecular pattern (DAMP)-mediated synovitis in OA remains limited. Dying chondrocytes release damage-associated molecular patterns—including reactive lipid-peroxidation aldehydes such as 4-hydroxynonenal and malondialdehyde, oxidized lipids, and mitochondrial DNA—that activate synovial macrophages and provoke synovitis, generating a fresh wave of IL-1β and TNF-α that re-enters chondrocytes to sustain iron overload and oxidative stress. The cytokine surge and GPX4 loss converge on a catabolic transcriptional program that upregulates MMP-13 and ADAMTS-5 while suppressing type II collagen and aggrecan, so that the structural hallmarks of OA—progressive loss of collagen and proteoglycan—emerge as downstream consequences of unchecked ferroptosis. Biomechanical cues feed the same circuit: excessive mechanical loading opens the Piezo1 mechanosensitive channel, and the resulting calcium influx lowers GSH and GPX4 to precipitate ferroptosis preferentially in the loaded zones of cartilage. The net effect is a vicious cycle in which iron overload, lipid peroxidation, antioxidant collapse, and inflammation reinforce one another to accelerate cartilage breakdown. The integrated mechanism is summarized in Figure 1.

**FIGURE 1:**
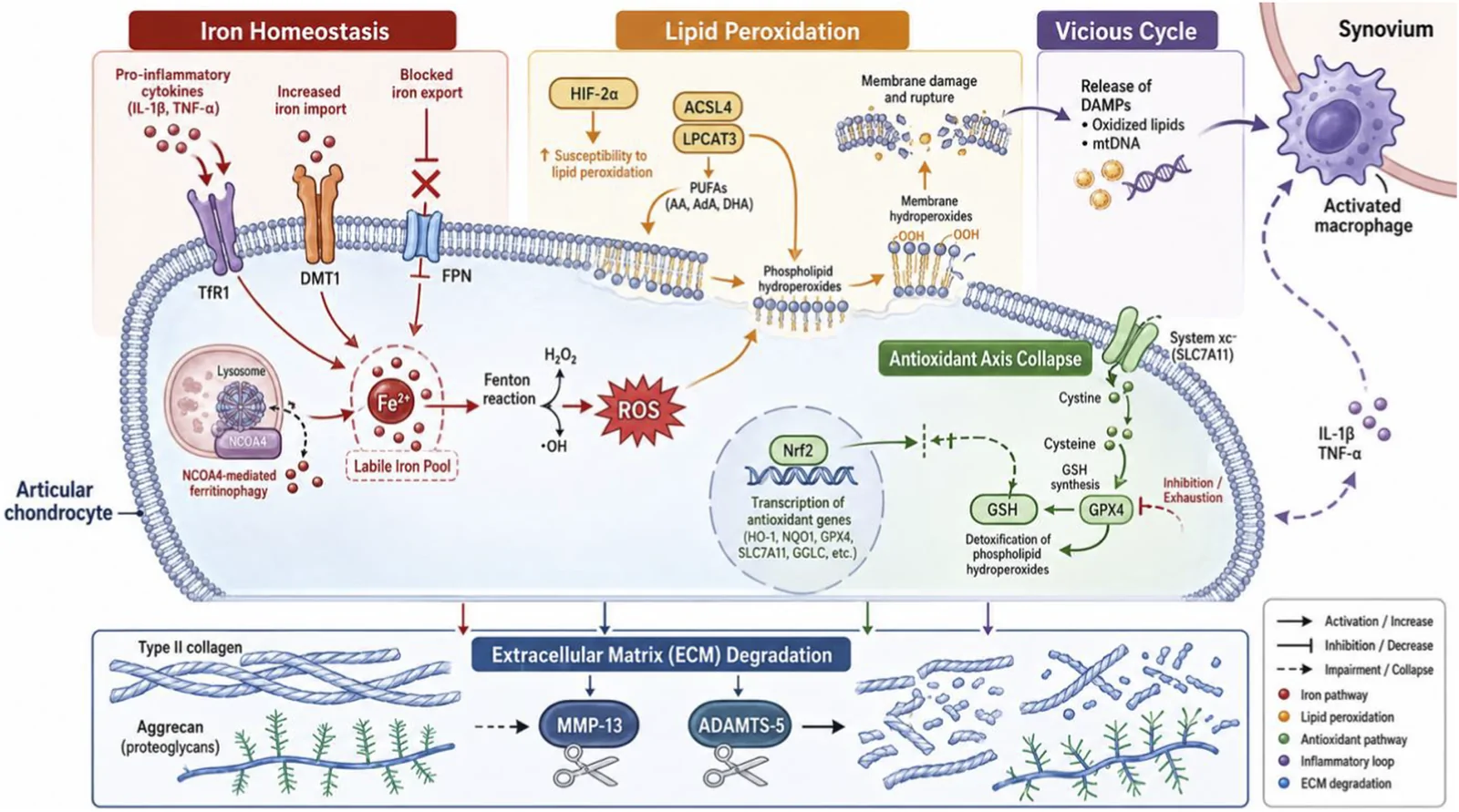
Mechanistic network of chondrocyte ferroptosis in osteoarthritis. Inflammation, increased iron import, impaired iron export, and NCOA4-mediated ferritinophagy expand the labile Fe^2+^ pool and drive Fenton chemistry and ACSL4/LPCAT3-dependent phospholipid peroxidation. Collapse of the system xc−–GSH–GPX4 axis permits membrane damage and ferroptotic death, while DAMP release, synovial macrophage activation, and MMP-13/ADAMTS-5-mediated matrix degradation establish a self-amplifying cartilage-destructive loop.

### 2.5 Pharmacological intervention strategies targeting ferroptosis

The therapeutic rationale for targeting ferroptosis in OA rests on its multiple druggable nodes and partial reversibility in experimental systems. Radical-trapping antioxidants such as ferrostatin-1, iron chelation, restoration of GPX4 activity, and reinforcement of complementary antioxidant pathways can reduce iron-dependent lipid peroxidation, preserve chondrocyte viability, and limit cartilage damage in preclinical models. Human cartilage studies further suggest that ferroptosis-related alterations vary with disease severity and may define stage-dependent therapeutic windows. These findings nevertheless require cautious interpretation. Prototype inhibitors have unfavorable pharmacokinetic properties, limited tissue selectivity, uncertain long-term safety, and no established clinical dosing strategy. Moreover, changes in reactive oxygen species, malondialdehyde, or GPX4 expression alone do not establish ferroptosis, because these readouts overlap with other forms of oxidative injury and regulated cell death. Future studies should combine mechanistically informative rescue experiments with direct assessment of iron-dependent phospholipid peroxidation, target engagement, extracellular matrix outcomes, and drug exposure in cartilage. These limitations support investigation of multi-target natural products while also explaining why delivery engineering is needed to concentrate active compounds at the chondrocyte and minimize off-target exposure.

## 3 Natural products as pharmacological modulators of chondrocyte ferroptosis

Natural products constitute a chemically diverse source of multi-target pharmacological modulators for chondrocyte ferroptosis. Unlike compounds designed around a single molecular target, many natural products concurrently influence iron transport, phospholipid peroxidation, mitochondrial redox homeostasis, cystine–glutathione metabolism, inflammatory signaling, and extracellular matrix turnover. The evidence base now encompasses tea polyphenols, curcuminoids, stilbenes, flavonoids, isoflavones, saponins, terpenoid glycosides, alkaloids, and related phenolic compounds. Nevertheless, the strength of evidence is uneven. Some agents have been evaluated using ferroptosis inducers or inhibitors together with iron, lipid peroxide, GPX4, SLC7A11, and ACSL4 readouts in chondrocytes and OA models, whereas others are supported mainly by antioxidant, anti-inflammatory, or general chondroprotective effects. Accordingly, the following sections distinguish direct anti-ferroptotic evidence from mechanistically adjacent or delivery-translatable findings and consider not only efficacy signals but also target specificity, model relevance, formulation feasibility, and safety. The principal compounds and their experimental evidence are summarized in Table 1.

**TABLE 1:** Natural products targeting chondrocyte ferroptosis in osteoarthritis.

Natural product	Source/chemical class	Major ferroptosis-related targets or pathways	Experimental models	Evidence level
Theaflavin-3,3′-digallate (TF3)	Black tea polyphenol/theaflavin derivative	Nrf2/GPX4 axis; lipid ROS suppression	Erastin-induced chondrocytes; experimental OA models	Direct chondrocyte ferroptosis evidence
Epigallocatechin-3-gallate (EGCG)	Green tea catechin/polyphenol	GPX4 preservation; Fe^2+^ and lipid peroxide reduction; ROS scavenging	Oxidative-stress-induced chondrocytes; DMM mouse OA model; EGCG–selenomethionine nanodrug	Direct evidence in nanodrug context
Curcumin	*Curcuma longa* L. [Zingiberaceae]/diarylheptanoid polyphenol	Nrf2/SLC7A11/GPX4; ACSL4; TfR1; Fe^2+^ homeostasis	Erastin-induced primary mouse chondrocytes	Direct chondrocyte ferroptosis evidence
Resveratrol	Grapes, berries, peanuts/stilbene polyphenol	G3BP1 stress granules/mTOR axis; ferroptosis-related protein regulation	High-fat-diet-induced obesity-related OA; IL-1β-treated chondrocytes	Direct OA ferroptosis evidence
Quercetin	Flavonol from fruits and vegetables	Nrf2/GPX4; HO-1; AMPK/Nrf2/GPX4	RSL3-induced chondrocytes; *in vitro* and *in vivo* OA models	Direct chondrocyte ferroptosis evidence
Baicalin	*Scutellaria baicalensis* Georgi [Lamiaceae]/flavone glycoside	Nrf2 signaling; Fe^2+^ and lipid peroxide control	IL-1β-induced human OA chondrocytes	Direct human OA chondrocyte evidence
Baicalein	*Scutellaria baicalensis* Georgi [Lamiaceae]/flavone aglycone	AMPKα/Nrf2/HO-1; GPX4-associated antioxidant defense	Chondrocyte ferroptosis models; experimental OA models	Direct chondrocyte ferroptosis evidence
Biochanin A	Red clover, chickpea/isoflavone	Nrf2/System Xc^−^/GPX4 axis; TfR1/FPN-mediated iron homeostasis	Iron-overload-associated knee OA; chondrocyte models	Direct iron-overload OA evidence
Icariin	Epimedium species/prenylated flavonol glycoside	SLC7A11/GPX4 axis	IL-1β-stimulated chondrocytes; experimental OA models	Direct chondrocyte ferroptosis evidence
Cardamonin	Alpinia species/chalcone flavonoid	p53 pathway; SLC7A11/GPX4-associated ferroptosis regulation	IL-1β-stimulated OA chondrocytes; cartilage degradation models	Direct chondrocyte ferroptosis evidence
Luteolin	Celery, green pepper, medicinal plants/flavone	AMPK–Nrf2 antioxidant signaling	H_2_O_2_-induced chondrocyte oxidative injury; OA progression models	Mechanistically adjacent evidence
Naringenin	Citrus fruits/flavanone	Oxidative stress suppression; iron-overload-associated cartilage injury	Iron-overload-induced OA models	Mechanistically adjacent iron-overload evidence
Formononetin	Legumes, Astragalus species/isoflavone	Anti-inflammatory and cartilage-protective signaling; cartilage targeting in PEG-FMN nanodrug	OA models treated with cartilage-targeting PEG-formononetin nanodrug	Delivery-translatable evidence
Sarsasapogenin	Anemarrhena asphodeloides, Smilax species/steroidal sapogenin	YAP1-dependent ferroptosis regulation	IL-1β-stimulated chondrocytes; DMM mouse OA model	Direct chondrocyte ferroptosis evidence
Asperosaponin VI	*Dipsacus asper* Wall. ex DC. [Caprifoliaceae]/triterpenoid saponin	Nrf2/GPX4/HO-1 axis; ACSL4 suppression	IL-1β-induced chondrocytes; experimental OA models	Direct chondrocyte ferroptosis evidence
Paeoniflorin	*Paeonia lactiflora* Pall. [Paeoniaceae]/monoterpene glycoside	p53/SLC7A11/GPX4 pathway	Iron-overload-induced OA; chondrocyte ferroptosis models	Direct iron-overload OA evidence
Paeonol	Paeonia species/phenolic compound	AMPK/Nrf2/GPX4 pathway; ACSL4-associated lipid peroxidation	IL-1β-induced chondrocytes; ferroptosis-related OA models	Direct chondrocyte ferroptosis evidence
Protopine	Corydalis and Fumaria species/isoquinoline alkaloid	Nrf2 signaling	tert-Butyl hydroperoxide-induced primary mouse chondrocytes	Direct chondrocyte ferroptosis evidence
Gossypol acetic acid	Cottonseed-derived polyphenolic aldehyde derivative	GPX4 methylation inhibition	OA chondrocyte ferroptosis models	Direct chondrocyte ferroptosis evidence, but safety-sensitive
Acetyl zingerone	Ginger/curcumin-inspired phenolic antioxidant	GPX4-associated programmed cell death regulation	OA chondrocyte programmed cell death models	Direct but broader PCD evidence

### 3.1 Polyphenols and phenolic derivatives

Polyphenols are among the most intensively investigated natural compounds for ferroptosis control because their phenolic hydroxyl groups allow them to quench free radicals, chelate transition metals, and remodel redox-sensitive signaling pathways. In the context of OA, tea-derived polyphenols provide representative examples. Theaflavin-3,3′-digallate (TF3), a black-tea polyphenol, inhibits erastin-induced ferroptosis in chondrocytes by activating the Nrf2/GPX4 signaling pathway, decreasing lipid peroxidation and improving ferroptosis-associated biochemical indices (Xu et al., 2022). Epigallocatechin-3-gallate (EGCG), the major catechin in green tea, has also been incorporated into supramolecular EGCG–selenomethionine nanodrugs, which reduce GPX4 inactivation, abnormal Fe^2+^ accumulation, and lipid peroxidation in oxidative-stress-induced chondrocytes and ameliorate OA progression after intra-articular administration in a destabilization of the medial meniscus model (Yu et al., 2024). Although this EGCG study already introduces a delivery system rather than free EGCG alone, it is particularly informative because it illustrates how a natural polyphenol can be converted into a self-assembled anti-ferroptotic nanomedicine.

Curcumin, a diarylheptanoid polyphenol from Curcuma longa, is another prototypical anti-ferroptotic compound. In erastin-induced primary mouse chondrocytes, curcumin reduced Fe^2+^ accumulation and lipid reactive oxygen species, restored SLC7A11 and GPX4, decreased ferroptosis-promoting markers such as ACSL4 and transferrin receptor 1, and activated Nrf2; Nrf2 silencing attenuated these protective effects, supporting an Nrf2-dependent mechanism (Zhou et al., 2023). This places curcumin at the intersection of antioxidant transcriptional activation and iron/lipid peroxide control. Resveratrol, a stilbene polyphenol abundant in grapes and berries, has recently been linked to ferroptosis inhibition in obesity-related OA. In high-fat-diet and IL-1β-associated OA settings, resveratrol mitigated chondrocyte ferroptosis through modulation of the G3BP1 stress granule/mTOR axis, highlighting a connection between metabolic stress, translational control, and ferroptotic vulnerability (He et al., 2026). These findings broaden the anti-ferroptotic scope of polyphenols beyond direct radical scavenging toward regulation of stress-granule dynamics and nutrient-sensing pathways.

Despite these advantages, polyphenols also expose the core translational dilemma of natural products. Curcumin and resveratrol are highly pleiotropic and biologically active, but their poor aqueous solubility, chemical instability, rapid metabolism, and limited systemic exposure severely restrict their therapeutic window. For OA, these limitations are magnified by the need to maintain effective concentrations within the joint cavity and to deliver compounds across the dense, avascular cartilage matrix to sparsely distributed chondrocytes. Thus, polyphenols are not only pharmacological leads but also paradigmatic examples of why nanoformulation is often indispensable.

### 3.2 Flavonoids and isoflavones

Flavonoids constitute a major class of natural ferroptosis modulators, largely because of their capacity to activate Nrf2-dependent antioxidant responses, suppress lipid peroxidation, and regulate mitochondrial homeostasis. Quercetin is one of the best-characterized examples. In RSL3-induced chondrocyte ferroptosis, quercetin increased GPX4, HO-1, and nuclear Nrf2, while reducing malondialdehyde and lipid ROS accumulation (Xu et al., 2023b). A subsequent *in vitro* and *in vivo* study further showed that quercetin attenuated OA symptoms by activating the AMPK/Nrf2/GPX4 pathway, suggesting that AMPK may serve as an upstream metabolic sensor linking quercetin to ferroptosis suppression (Dong et al., 2025). These results position quercetin as a representative flavonol capable of restoring both energy-redox coupling and GPX4-mediated phospholipid peroxide detoxification.

Baicalin and baicalein, two flavonoids derived from Scutellaria baicalensis, similarly target the Nrf2 antioxidant axis. Baicalin inhibits IL-1β-induced ferroptosis in human OA chondrocytes by activating Nrf2 signaling, reducing Fe^2+^ accumulation, lipid peroxidation, and matrix catabolic responses (Liu et al., 2024). Baicalein limits OA development by activating AMPKα/Nrf2/HO-1 signaling, suppressing chondrocyte ferroptosis, and improving cartilage degeneration in experimental OA models (Wan et al., 2023a). These two structurally related flavonoids demonstrate how subtle differences in glycosylation and physicochemical properties may influence cellular uptake, stability, and delivery requirements, even when their convergent pharmacological output is Nrf2-driven ferroptosis inhibition.

Isoflavones also show strong relevance to iron and redox control. Biochanin A protects against iron-overload-associated knee OA by regulating iron levels and activating the Nrf2/system xc−/GPX4 axis; it reduces iron accumulation, ROS production, lipid ROS, and mitochondrial damage, while suppressing transferrin receptor 1 and promoting ferroportin expression (He et al., 2023). Icariin, a prenylated flavonol glycoside from Epimedium, inhibits IL-1β-induced chondrocyte ferroptosis by enhancing SLC7A11 and GPX4 expression and ameliorates OA *in vivo* (Xiao et al., 2024). Cardamonin, a chalcone from Alpinia species, alleviates chondrocyte inflammation and cartilage degradation by inhibiting ferroptosis through the p53 pathway (Gong et al., 2023). Together, these studies indicate that flavonoids do not act through a single shared target; rather, they converge on ferroptosis suppression through several regulatory routes, including AMPK/Nrf2/GPX4, Nrf2/system xc−/GPX4, SLC7A11/GPX4, iron export/import balance, and p53-associated cystine metabolism.

Some flavonoids are better interpreted as delivery-relevant or mechanistically adjacent candidates. Luteolin attenuates oxidative injury and OA progression by activating AMPK–Nrf2 signaling in chondrocytes, but direct ferroptosis endpoints have not been validated as comprehensively as for quercetin or baicalein (Zhou et al., 2022). Naringenin protects against iron-overload-induced OA by suppressing oxidative stress, supporting relevance to iron-driven cartilage injury without independently confirming ferroptosis as the decisive mechanism (Pan et al., 2022). Formononetin has been incorporated into a collagen-binding, cartilage-targeted PEG nanodrug, demonstrating that an isoflavone scaffold can be engineered for improved cartilage retention and OA therapy (Xiong et al., 2021). Thus, direct anti-ferroptotic evidence is strongest for quercetin, baicalin, baicalein, biochanin A, icariin, and cardamonin, whereas luteolin, naringenin, and formononetin provide mechanistically adjacent or delivery-translatable evidence.

### 3.3 Saponins, terpenoid glycosides, alkaloids, and other natural products

Beyond polyphenols and flavonoids, several structurally distinct natural compounds suppress chondrocyte ferroptosis through mechanisms that broaden the druggable landscape. Sarsasapogenin, a steroidal sapogenin, inhibits YAP1-dependent chondrocyte ferroptosis and alleviates OA progression in both IL-1β-stimulated chondrocytes and destabilization of the medial meniscus models (Chen et al., 2023a). This is particularly important because it links ferroptosis to mechanotransduction and transcriptional regulation, rather than only to canonical antioxidant pathways. Asperosaponin VI, a triterpenoid saponin from Dipsacus asper, suppresses chondrocyte ferroptosis and ameliorates OA through the Nrf2/GPX4/HO-1 pathway, decreasing ACSL4 and inflammatory mediators while restoring cartilage matrix homeostasis (Zhang et al., 2025). These saponin-related studies suggest that compounds traditionally viewed as anti-inflammatory or bone-protective agents can also directly regulate ferroptotic death programs.

Paeoniflorin, a monoterpene glycoside from Paeonia lactiflora, mitigates iron-overload-induced OA by suppressing chondrocyte ferroptosis through the p53/SLC7A11/GPX4 pathway (Wu et al., 2025). This finding is mechanistically relevant because p53 can repress SLC7A11 and sensitize cells to ferroptosis; paeoniflorin therefore acts upstream of cystine import and GSH-dependent lipid peroxide detoxification. Paeonol, another bioactive compound from Paeonia species, ameliorates IL-1β-induced ferroptosis and inflammation in chondrocytes through the AMPK/Nrf2/GPX4 pathway (Gong et al., 2025). These two Paeonia-derived agents exemplify how related medicinal plant sources may yield chemically different compounds that converge on ferroptosis suppression through distinct upstream regulators.

Alkaloids and other phenolic derivatives further diversify the anti-ferroptotic natural-product repertoire. Protopine, an isoquinoline alkaloid, protects primary mouse chondrocytes from tert-butyl hydroperoxide-induced ferroptosis by activating Nrf2 signaling (Chen et al., 2024). Gossypol acetic acid alleviates chondrocyte ferroptosis in OA by inhibiting GPX4 methylation, suggesting an epigenetic route to restore GPX4 function (Shang et al., 2025). Acetyl zingerone ameliorates experimental OA by modulating several programmed cell-death pathways; GPX4-associated changes suggest a ferroptosis-related component, but the evidence is broader than a ferroptosis-specific mechanism (Chen et al., 2023b). Collectively, these studies indicate that natural compounds may engage transcriptional, metabolic, and epigenetic control nodes rather than acting solely as nonspecific antioxidants.

### 3.4 Pharmacokinetic, standardization, and translational limitations

Despite compelling mechanistic signals, natural products face several pharmacological barriers before they can be considered viable OA therapeutics. Many anti-ferroptotic candidates, including curcumin, resveratrol, quercetin, baicalein, and sarsasapogenin, have low aqueous solubility and are difficult to formulate at reproducible concentrations. Phenolic compounds may also undergo oxidation or degradation, while extensive glucuronidation, sulfation, and rapid clearance can produce low or short-lived systemic exposure; curcumin and resveratrol are representative examples (Liu et al., 2020; Walle, 2011). Consequently, concentrations used in cell culture may not correspond to achievable concentrations in plasma, synovial fluid, cartilage, or chondrocytes. Few studies establish dose–exposure–response relationships, compare oral and intra-articular administration, or demonstrate that the proposed ferroptosis target is engaged at the site of action.

Translation is also limited by inconsistency in compound identity, purity, extraction or synthesis procedures, and reporting of batch characteristics. Apparent multi-target activity may be advantageous, but it can also obscure the contribution of individual pathways and increase the risk of off-target or concentration-dependent effects. Safety requires compound-specific evaluation: a history of dietary or traditional use does not establish the safety of purified compounds, high local doses, repeated intra-articular administration, or nanocarrier-associated exposure. Future studies should report physicochemical characterization, stability, purity, release behavior, pharmacokinetics, tissue distribution, maximum tolerated exposure, and predefined ferroptosis-specific endpoints. These requirements separate intrinsic pharmacological limitations of the active compound from the joint-specific transport barriers discussed in Section 4 and provide the basis for rational nanodelivery rather than formulation by trial and error.

## 4 Joint-specific barriers to intra-articular delivery

Intra-articular (IA) administration is attractive for OA therapy because it delivers drugs directly into the diseased joint and can reduce systemic exposure. Local injection, however, does not guarantee effective delivery to chondrocytes. A therapeutic compound must remain in the joint cavity, avoid rapid synovial and lymphatic clearance, penetrate the dense cartilage extracellular matrix (ECM), reach sparsely distributed chondrocytes, enter cells, and maintain sufficient intracellular exposure to regulate iron metabolism, lipid peroxidation, and the Nrf2/system xc−/GSH/GPX4 antioxidant network (Evans et al., 2014; Gerwin et al., 2006; Larsen et al., 2008). These sequential barriers make IA therapy a tissue-transport and pharmacokinetic challenge rather than simply a route of administration.

### 4.1 Rapid joint clearance limits local exposure

The synovial cavity is a dynamically exchanged anatomical space rather than a closed depot. Small molecules are cleared mainly through synovial capillaries, whereas macromolecules and particulate systems are influenced by lymphatic drainage and synovial interstitial transport. Classical kinetic studies showed that solutes injected into synovial fluid disappear in a molecular-size-dependent manner, with many low-molecular-weight agents cleared within minutes to hours (Owen et al., 1994). This is problematic for anti-ferroptotic natural products such as curcumin, resveratrol, quercetin, baicalein, paeonol, and sarsasapogenin, whose poor solubility, instability, and limited tissue permeability already reduce effective exposure.

OA further complicates this pharmacokinetic environment. Synovial inflammation alters vascular permeability, lymphatic drainage, and synovial tissue transport, while synovial fluid composition can affect nanoparticle stability, diffusion, and cellular uptake (Sterner et al., 2016; Guang et al., 2021; Siefen et al., 2021; Cao et al., 2022). Therefore, increasing the dose of free drug is unlikely to solve the delivery problem and may increase synovial off-target effects. A rational formulation must instead prolong joint residence, protect the cargo, and regulate the rate and direction of drug transport toward cartilage.

### 4.2 Cartilage ECM forms a dense and negatively charged penetration barrier

Articular cartilage is avascular, aneural, and alymphatic, and is composed mainly of water, type II collagen, aggrecan-rich proteoglycans, and sparsely distributed chondrocytes (Sophia Fox et al., 2009). The collagen II network provides tensile strength, whereas aggrecan and glycosaminoglycan chains generate a high fixed negative charge density and osmotic swelling pressure. These structural features are essential for load-bearing function but severely restrict drug penetration (Maldonado and Nam, 2013).

Transport through cartilage depends on molecular size, charge, matrix affinity, and disease state. Large neutral or anionic molecules generally diffuse poorly and may remain near the superficial zone. In contrast, appropriately sized cationic carriers can exploit electrostatic interactions with negatively charged glycosaminoglycans to enhance cartilage uptake and retention (Bajpayee and Grodzinsky, 2017). Avidin-based studies demonstrated that small positively charged carriers can penetrate cartilage and achieve prolonged intratissue residence, supporting the concept of cartilage as an electrostatically addressable depot (Bajpayee et al., 2014). However, charge-mediated targeting requires careful optimization. Excessive cationic charge may cause superficial trapping, cytotoxicity, matrix perturbation, or nonspecific synovial uptake. Cationic peptide carrier studies therefore emphasize that net charge, hydrophobicity, and molecular size must be balanced to achieve deep cartilage penetration while preserving chondrocyte viability (Ve et al., 2019; Vedadghavami et al., 2020).

### 4.3 Chondrocytes are sparse and difficult intracellular targets

Even when a drug enters cartilage, it still needs to reach chondrocytes embedded within lacunae and surrounded by pericellular matrix. This is particularly important for ferroptosis-targeted therapy because the key pharmacological events occur intracellularly, including restoration of GPX4 activity, maintenance of GSH, regulation of SLC7A11, suppression of ACSL4-dependent lipid peroxidation, limitation of labile Fe^2+^ accumulation, and mitochondrial protection. Thus, tissue accumulation alone is insufficient; effective systems must also promote chondrocyte uptake and intracellular release.

This distinction explains why successful OA nanomedicine often requires several levels of targeting: joint retention, cartilage binding, ECM penetration, chondrocyte uptake, and controlled intracellular release. Cartilage-penetrating framework nucleic acid nanoparticles improved both cartilage penetration and chondrocyte uptake (Huang et al., 2025). Similarly, PEGylated PAMAM nanocarriers enhanced cartilage binding, deep penetration, joint residence, and the efficacy of growth-factor treatment compared with free cargo (Geiger et al., 2018). Although the payloads differ from the natural products emphasized here, these studies provide transferable design principles for intracellular ferroptosis-directed delivery. These sequential barriers and corresponding nanomedicine solutions are summarized in Figure 2.

**FIGURE 2:**
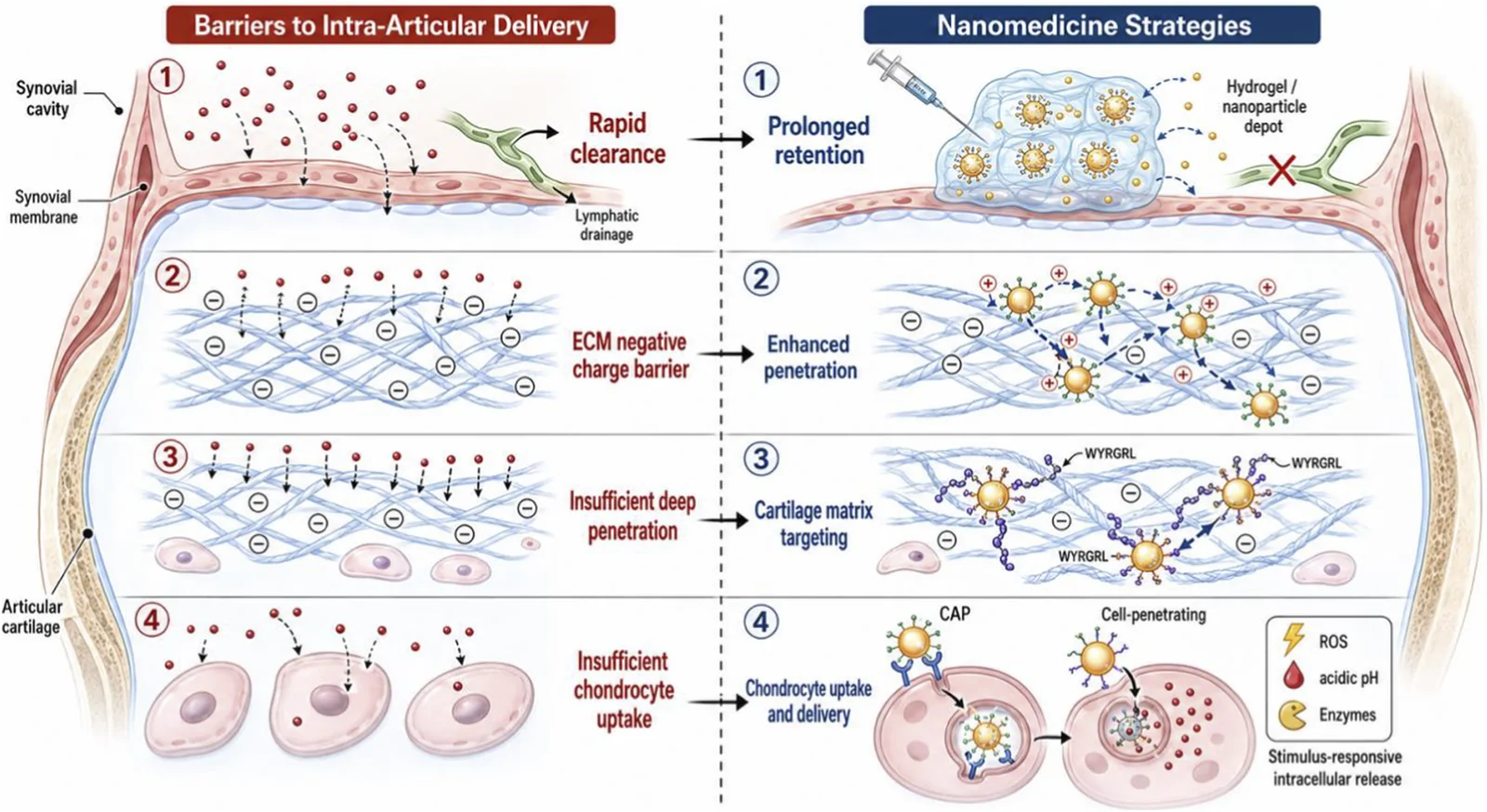
Barriers to intra-articular delivery and corresponding nanomedicine strategies. Rapid synovial and lymphatic clearance, the dense negatively charged cartilage matrix, limited deep penetration, and poor chondrocyte uptake restrict free-drug exposure. Sustained-release depots, size- and charge-optimized carriers, collagen II-binding ligands such as WYRGRL, chondrocyte-affinity peptide (CAP), and ROS-, pH-, or enzyme-responsive systems can improve joint retention, cartilage localization, cellular uptake, and intracellular release.

## 5 Pharmacological optimization of natural products through nanodelivery

The barriers described above indicate that an effective intra-articular system must do more than encapsulate a natural product. It should improve solubility and chemical stability, provide a reproducible release profile, prolong joint residence, penetrate or bind cartilage, facilitate chondrocyte uptake, and maintain intracellular exposure long enough to modulate ferroptosis-related targets. Current OA nanomedicines include polymeric nanoparticles, lipid-based carriers, hydrogels, biomimetic vesicles, and inorganic or hybrid materials (Li et al., 2021; Liang et al., 2024; Wen et al., 2023). For pharmacological optimization, carrier selection should be driven by the dominant liability of the active compound and the intended site of action: hydrophobic compounds require solubilization, unstable molecules require protection, rapidly cleared drugs benefit from sustained-release depots, and intracellular modulators require systems capable of reaching chondrocytes without excessive synovial sequestration.

### 5.1 Main nanodelivery platforms

Polymeric nanoparticles and polymer–drug conjugates are among the most adaptable platforms for OA delivery. PLGA, PEGylated systems, PAMAM dendrimers, and polymeric micelles can solubilize hydrophobic natural products, reduce burst release, and provide tunable degradation. A collagen-binding PEG–formononetin nanodrug illustrates this strategy: PEGylation improves pharmaceutical behavior, while ligand-mediated binding enhances cartilage retention. Dendrimer-based systems are also attractive because their small size, high functional-group density, and adjustable charge favor cartilage interaction and ligand modification; PEGylated PAMAM nanocarriers have shown improved cartilage penetration and therapeutic retention.

Lipid-based carriers, including liposomes, lipid nanoparticles, and lipid–polymer hybrids, are suitable for hydrophobic natural products and membrane-associated targets. This is particularly relevant to ferroptosis, which is executed at the level of lipid membranes. A pH-responsive lipid nanoparticle loaded with urolithin A improved mitochondrial function and inhibited ferroptosis in OA models, supporting the value of lipid platforms for natural compounds acting on mitophagy–ferroptosis crosstalk (Yi et al., 2025). Lipid systems are also easy to modify with PEG, peptides, hyaluronic acid, or other ligands, although their stability in synovial fluid and resistance to mechanical stress still require optimization.

Hydrogels and self-assembled systems mainly address the residence-time problem. Thermosensitive or injectable hydrogels can form local depots after IA injection and provide sustained release. Thermosensitive hydrogel-mediated quercetin delivery attenuated cartilage degradation in a rat OA model (Mok et al., 2020). Supramolecular EGCG–selenomethionine nanodrugs further show that natural products can participate directly in self-assembly and function as both pharmacological cargo and structural components (Yu et al., 2024). Hydrogels are useful for prolonged exposure, but their network structure may limit deep cartilage penetration unless they release smaller nanoparticles or molecular species.

Biomimetic systems, including extracellular vesicles and cell-membrane-coated nanoparticles, aim to improve biocompatibility and pathological-site recognition. Targeting peptide-modified engineered exosomes encapsulated in injectable hydrogels have shown enhanced cartilage-directed delivery and OA protection (Wan et al., 2023b). These systems are attractive because vesicle membranes naturally carry adhesion molecules and signaling lipids, but production, purification, cargo loading, and batch consistency remain challenging.

Inorganic and hybrid nanomaterials provide functions beyond delivery. Cerium oxide nanoparticles can scavenge ROS through enzyme-mimetic redox cycling, and WYRGRLGK peptide-modified cerium oxide nanoparticles achieved prolonged cartilage residence, improved penetration, and protection against oxidative cartilage injury (Zhuang et al., 2024). MXene nanosheets have also been used to enhance quercetin delivery and chondroprotection in experimental OA, with stronger inhibition of ferroptosis than free quercetin (Gan et al., 2025). These systems are particularly relevant when the carrier itself contributes antioxidant or anti-ferroptotic activity, but long-term biodegradation and joint safety require further evaluation.

### 5.2 Cartilage- and chondrocyte-targeting ligand strategies

Targeting ligands can be divided into cartilage matrix-targeting ligands, chondrocyte-targeting ligands, and inflammation/synovium-directed ligands. Among them, peptide ligands deserve special attention because they are small, chemically tunable, easy to conjugate, and compatible with polymers, lipids, inorganic nanoparticles, hydrogels, and extracellular vesicles.

The collagen type II-binding peptide WYRGRL is one of the most widely used cartilage matrix-targeting peptides. Rothenfluh et al. first demonstrated that WYRGRL-functionalized polymer nanoparticles improved intra-articular cartilage targeting and retention (Rothenfluh et al., 2008). Since type II collagen is abundant in cartilage ECM, WYRGRL modification has subsequently been used in multiple platforms. In a dual-drug nanoplatform, WYRGRL was conjugated to MOF-decorated mesoporous polydopamine nanoparticles, enabling cartilage targeting, imaging, and improved OA protection (Xue et al., 2021). WYRGRLGK-modified cerium oxide nanoparticles further confirmed the value of collagen II-targeting peptides for combining cartilage localization with ROS-scavenging nanozyme activity.

Chondrocyte affinity peptide (CAP) represents a more cell-directed strategy. CAP was identified by phage display and used to modify PEI/DNA complexes, resulting in higher cartilage accumulation and more specific chondrocyte uptake than scrambled peptide controls (Pi et al., 2011). CAP-modified PEGylated PAMAM conjugates further improved cellular uptake, cartilage retention, and *in vivo* penetration, suggesting that CAP is useful when intracellular delivery is required (Hu et al., 2018). This is highly relevant to ferroptosis-targeted therapy because most regulatory nodes, including GPX4, SLC7A11, ACSL4, Nrf2, labile Fe^2+^, and mitochondrial redox control, are intracellular.

Cationic peptide carriers offer another strategy based on electrostatic interaction with negatively charged glycosaminoglycans. They can enhance cartilage penetration and retention, but charge density must be carefully balanced to avoid superficial trapping and cytotoxicity Anti-inflammatory peptides such as KAFAK can also be delivered by thermoresponsive nanoparticles; KAFAK-loaded hollow PEGylated poly (NIPAM) nanoparticles reduced IL-1β-induced inflammatory responses in cartilage explants, illustrating that peptides may act both as targeting/penetrating modules and as therapeutic agents (McMasters et al., 2017).

Non-peptide ligands provide complementary advantages. Hyaluronic acid (HA) can interact with CD44 on chondrocytes and synoviocytes; self-assembled HA nanoparticles achieved prolonged joint retention and suppressed CD44/NF-κB-associated catabolic signaling in experimental OA (Kang et al., 2021). Chondroitin sulfate can also serve as a coating or cartilage-related targeting component: chondroitin sulfate-coated ROS-scavenging nanoparticles protected cartilage *in vivo* (Wang et al., 2023), whereas chondrocyte-targeted chondroitin sulfate–N-acetylcysteine nanoparticles maintained glutathione homeostasis and inhibited ferroptosis (Wang et al., 2026). Compared with peptides, HA and chondroitin sulfate are more biomimetic, but their distribution and apparent specificity may vary with disease stage, receptor expression, and synovial uptake.

An effective targeting design is unlikely to depend on a single mechanism. For natural products directed at chondrocyte ferroptosis, a rational system may combine cartilage-matrix anchoring, controlled penetration, chondrocyte uptake, and stimulus-responsive release. Peptide functionalization is particularly useful because it can be added modularly to different carrier backbones. These design principles provide the basis for evaluating the integrated natural product–nanodelivery systems discussed in the next section.

## 6 Integrated natural product–nanodelivery strategies: representative evidence

Although many natural products suppress chondrocyte ferroptosis *in vitro*, only a smaller group of studies has integrated all three elements required for disease-modifying OA therapy: an anti-ferroptotic natural product, an intra-articular nanodelivery platform, and validation in cartilage or OA models. These integrated studies are particularly valuable because they show how delivery engineering can convert mechanistically active but pharmaceutically limited compounds into joint-localized therapeutics.

Polyphenol-based nanodrugs provide the clearest examples. EGCG–selenomethionine nanodrugs reduced Fe^2+^ accumulation, lipid peroxidation, and GPX4 inactivation while improving cartilage pathology after IA injection (Yu et al., 2024). EGCG–cysteine nanoparticles similarly inhibited ROS and Fe^2+^ accumulation, lipid peroxidation, and ferroptosis-related cartilage metabolic disturbance in H_2_O_2_-stimulated chondrocytes and DMM-induced OA models (Lin et al., 2026). These studies show that polyphenols can function not only as payloads but also as self-assembling structural units, simplifying formulation while preserving redox-regulating activity.

Curcumin-based systems further illustrate the value of nanocarrier-enabled multi-target regulation. Curcumin-loaded nanoparticles suppressed chondrocyte ferroptosis by reducing ROS, Fe^2+^, ACSL4, mitochondrial injury, and lipid peroxidation, while restoring cartilage anabolic markers (Sheng et al., 2025). A macrophage membrane-coated curcumin nanosponge achieved broader synergy by combining curcumin-mediated ferroptosis inhibition with membrane-mediated inflammatory cytokine neutralization; this system reduced Fe^2+^, ACSL4, MDA, and lipid peroxidation while increasing SLC7A11 and GPX4, leading to improved cartilage protection *in vivo* (Xu et al., 2024). These findings support a key design principle: for OA, anti-ferroptotic efficacy is strengthened when lipid peroxidation control is coupled with suppression of synovial inflammation.

Other natural product-based systems expand this strategy beyond polyphenols. MXene-mediated quercetin delivery produced stronger chondroprotective and anti-ferroptotic effects than free quercetin in experimental OA (Gan et al., 2025). A pH-responsive lipid nanoparticle delivering urolithin A connected mitochondrial quality control with ferroptosis suppression, suggesting that mitophagy–ferroptosis crosstalk can be exploited through microenvironment-responsive release (Yi et al., 2025). Baicalin-based functional polymers incorporated into dynamic reversible networks prolonged local retention and reduced cartilage degeneration and synovial inflammation; however, this study is better classified as delivery-translatable evidence than as direct confirmation of ferroptosis inhibition (Yang et al., 2025).

Several non-natural-product or carrier-dominant systems provide adjacent evidence for future formulation design. Chondrocyte-targeted chondroitin sulfate–N-acetylcysteine nanoparticles restored glutathione homeostasis, inhibited ferroptosis, and protected cartilage under mechanical stress (Wang et al., 2026). Cartilage-targeted cerium oxide nanoparticles prolonged cartilage residence and used nanozyme-mediated ROS scavenging to protect cartilage (Zhuang et al., 2024). Bioengineered chondrocyte membrane-camouflaged rosiglitazone liposomes enhanced cartilage penetration, prolonged joint retention, and impeded chondrocyte ferroptosis by regulating iron homeostasis and lipid peroxidation (Cao et al., 2024). Although these systems do not all use natural products as payloads, their targeting, retention, and intracellular-delivery principles are transferable to natural anti-ferroptotic agents.

Overall, current integrated studies suggest three practical design routes. First, self-assembled natural product nanodrugs, represented by EGCG-based systems, minimize excipient complexity and preserve intrinsic redox activity. Second, carrier-enhanced delivery of hydrophobic compounds, represented by curcumin and quercetin systems, improves bioavailability and strengthens inhibition of lipid peroxidation. Third, biomimetic or targeted nanoplatforms add tissue selectivity and anti-inflammatory synergy, which is essential because ferroptosis and synovitis reinforce each other in OA. However, the field remains early: only a limited number of studies simultaneously demonstrate natural-product loading, cartilage/chondrocyte targeting, ferroptosis inhibition, and *in vivo* structural protection. Future studies should therefore compare free drug, non-targeted carrier, targeted carrier, and unloaded carrier controls in the same model to distinguish payload-driven, carrier-driven, and targeting-driven effects. Representative compound–carrier–target relationships are illustrated in Figure 3, whereas the delivery features, experimental models, and principal pharmacological findings of the available integrated systems are summarized in Table 2.

**FIGURE 3:**
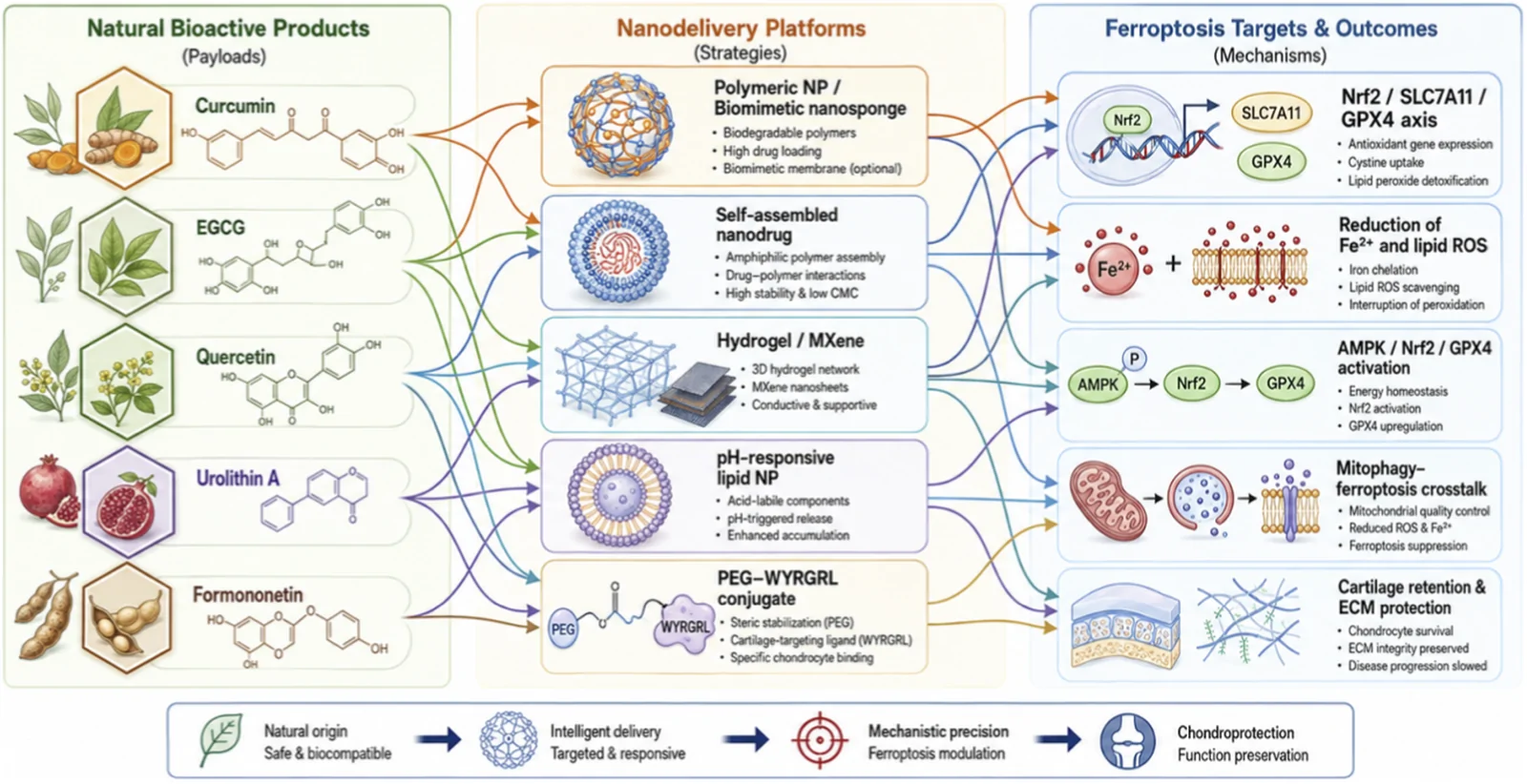
Integrated pharmacological design framework for natural product-based nanomedicine targeting chondrocyte ferroptosis. Representative natural products are matched with polymeric or biomimetic nanoparticles, self-assembled nanodrugs, hydrogels or MXene systems, pH-responsive lipid nanoparticles, and PEG–WYRGRL conjugates. These strategies aim to restore Nrf2/SLC7A11/GPX4 signaling, reduce Fe^2+^ and lipid ROS, regulate AMPK and mitophagy–ferroptosis crosstalk, and enhance cartilage retention and extracellular-matrix protection. The illustrated pairings are representative rather than exclusive.

**TABLE 2:** Representative natural product–nanodelivery systems evaluated in osteoarthritis models.

Natural product	Delivery platform	Targeting/release feature	Model	Main pharmacological finding	Ref.
EGCG	EGCG–selenomethionine supramolecular nanodrug	Self-assembly; IA administration	Oxidative-stress chondrocytes; DMM mouse OA	Reduced Fe^2+^ accumulation, lipid peroxidation, and GPX4 inactivation	(Yu et al., 2024)
Formononetin	PEG–formononetin with collagen-binding peptide	Cartilage-matrix targeting	Experimental OA	Improved cartilage retention and chondroprotection; direct ferroptosis evidence not established	(Xiong et al., 2021)
Urolithin A	pH-responsive lipid nanoparticles	Acid-responsive release	OA chondrocytes and animal model	Linked mitophagy regulation with ferroptosis suppression	(Yi et al., 2025)
Quercetin	Thermosensitive hydrogel	Sustained IA depot	Rat OA	Reduced cartilage degradation; ferroptosis-specific mechanism not directly established	(Mok et al., 2020)
Quercetin	MXene-mediated nanocarrier	Carrier-enhanced cellular delivery	Experimental OA	Stronger chondroprotective and anti-ferroptotic effects than free quercetin	(Gan et al., 2025)
EGCG	EGCG–cysteine self-assembled nanodrug	Self-assembly	H_2_O_2_-stimulated chondrocytes; DMM mouse OA	Reduced ROS, Fe^2+^ accumulation, lipid peroxidation, and metabolic disturbance	(Lin et al., 2026)
Curcumin	Curcumin-loaded nanoplatform	Enhanced exposure and cellular delivery	Experimental OA	Reduced ACSL4, mitochondrial injury, and lipid peroxidation; restored anabolic markers	(Sheng et al., 2025)
Curcumin	Macrophage membrane-coated nanosponge	Biomimetic inflammatory-microenvironment targeting	Experimental OA	Combined cytokine neutralization with SLC7A11/GPX4 restoration and ferroptosis suppression	(Xu et al., 2024)
Baicalin	Functional polymer in a dynamic reversible network	Local retention and cell-interactive delivery	Experimental OA	Reduced synovitis and cartilage degeneration; ferroptosis evidence indirect	(Yang et al., 2025)

Direct ferroptosis evidence requires iron-dependent lipid-peroxidation readouts together with mechanistic perturbation or rescue; entries described as indirect should not be interpreted as confirmed ferroptosis inhibition.

## 7 Discussion

Collectively, available evidence supports chondrocyte ferroptosis as a pharmacologically accessible component of OA pathobiology and identifies natural products as a versatile source of multi-target modulators. The strongest direct evidence centers on restoration of the Nrf2/SLC7A11/GPX4 antioxidant network, suppression of ACSL4-dependent phospholipid peroxidation, and correction of iron-handling abnormalities. However, the literature is disproportionately focused on a limited set of redox markers and signaling pathways. Many studies infer ferroptosis from oxidative stress, inflammation, or GPX4 expression without sufficiently demonstrating iron-dependent phospholipid peroxidation or mechanistic rescue (Brandt et al., 2025). Antioxidant or chondroprotective activity should therefore not automatically be classified as ferroptosis inhibition.

Nanodelivery offers a rational means of converting poorly soluble or rapidly cleared natural products into joint-localized pharmacological systems. Promising designs combine sustained IA exposure with cartilage-matrix binding, controlled penetration, chondrocyte uptake, and disease-responsive release. Nevertheless, improved efficacy cannot automatically be attributed to the natural product because carriers may independently scavenge reactive oxygen species, alter inflammation, or change cellular uptake. Future studies should include free-drug, unloaded-carrier, non-targeted-carrier, and targeted-carrier controls, together with quantitative pharmacokinetics and tissue distribution. Repeated-dose biocompatibility, biodegradation, immunogenicity, manufacturing reproducibility, sterilization, and scale-up are especially important for biomimetic, inorganic, and hybrid systems and are common barriers to clinical nanomedicine translation (Galluzzi et al., 2018).

Clinical translation also requires moving beyond uniform treatment of experimental OA. OA is heterogeneous across structural, inflammatory, mechanical, metabolic, and pain-dominant phenotypes (Anselmo and Mitragotri, 2019; Katz et al., 2021), and ferroptosis-related activity may vary with disease stage, mechanical loading, synovitis, metabolic status, and systemic or local iron handling. Biomarkers reflecting phospholipid peroxidation, iron dysregulation, antioxidant capacity, or cartilage target engagement may help identify patients most likely to benefit and define appropriate therapeutic windows. Before early-phase trials, the field needs standardized natural-product characterization, dose–response and exposure–response analyses, clinically feasible formulations, large-animal validation, and comparison with accepted OA treatments. Repeated IA dosing should be evaluated for both local joint safety and systemic exposure.

## 8 Conclusion

In conclusion, natural products and nanodelivery are complementary rather than interchangeable components of ferroptosis-targeted OA therapy. Natural products provide network-level pharmacology, whereas engineered carriers determine whether that pharmacology reaches cartilage and chondrocytes at an effective and tolerable exposure. A credible disease-modifying strategy will require rigorous confirmation that ferroptosis is the relevant mechanism, rational matching of compound liabilities with carrier design, and validation in biologically and clinically relevant OA phenotypes. Integrating target biology, pharmacological evidence, and delivery engineering may move the field from proof-of-concept antioxidant effects toward reproducible, mechanism-based, and potentially translatable therapies.
